# Structural Changes, Biological Consequences, and Repurposing of Colchicine Site Ligands

**DOI:** 10.3390/biom13050834

**Published:** 2023-05-14

**Authors:** Felipe Montecinos, Dan L. Sackett

**Affiliations:** 1 Protein Expression Laboratory, National Institute of Arthritis and Musculoskeletal and Skin Diseases, National Institutes of Health, Bethesda, MD 20892, USA; 2 Division of Basic and Translational Biophysics, *Eunice Kennedy Shriver* National Institute of Child Health and Human Development, National Institutes of Health, Bethesda, MD 20892, USA; sackettd@mail.nih.gov

**Keywords:** microtubule, tubulin, colchicine, benzimidazole, repurposing

## Abstract

Microtubule-targeting agents (MTAs) bind to one of several distinct sites in the tubulin dimer, the subunit of microtubules. The binding affinities of MTAs may vary by several orders of magnitude, even for MTAs that specifically bind to a particular site. The first drug binding site discovered in tubulin was the colchicine binding site (CBS), which has been known since the discovery of the tubulin protein. Although highly conserved throughout eukaryotic evolution, tubulins show diversity in their sequences between tubulin orthologs (inter-species sequence differences) and paralogs (intraspecies differences, such as tubulin isotypes). The CBS is promiscuous and binds to a broad range of structurally distinct molecules that can vary in size, shape, and affinity. This site remains a popular target for the development of new drugs to treat human diseases (including cancer) and parasitic infections in plants and animals. Despite the rich knowledge about the diversity of tubulin sequences and the structurally distinct molecules that bind to the CBS, a pattern has yet to be found to predict the affinity of new molecules that bind to the CBS. In this commentary, we briefly discuss the literature evidencing the coexistence of the varying binding affinities for drugs that bind to the CBS of tubulins from different species and within species. We also comment on the structural data that aim to explain the experimental differences observed in colchicine binding to the CBS of β-tubulin class VI (TUBB1) compared to other isotypes.

## 1. Introduction

Small molecules bind to proteins via reversible and irreversible (covalent) pathways. Reversible interactions cover an affinity range (K_d_) from mM (many enzymes and their substrates) to fM (biotin–avidin K_d_ of ~10^−15^ M, for example) [1,2]. A given ligand can have very different affinities for similar targets in different cells owing to differences between orthologs (nominally the same protein in different organisms) or paralogs (different proteins in the same organism related by gene duplication, referred to as isotypes).

Microtubule-Targeting Agents (MTAs), which all bind to tubulin, share this pattern of binding affinity variation, covering many orders of magnitude. We discuss this in the context of differences between tubulin orthologs (i.e., inter-species structural variations between nominally the same protein in different species) and tubulin paralogs (i.e., within-species variations, between closely related proteins separated by gene duplications; i.e., between isotypes). Several MTA binding sites on tubulin have been and continue to be targeted by research for novel therapeutics [3,4]. We will primarily consider MTAs that bind at the colchicine binding site (Colchicine Binding Site Inhibitors, or CBSIs). Colchicine was the first defined ligand binding site on tubulin, and its binding was definitive: tubulin was known as the “Colchicine Binding Protein” when first discovered [5,6]. This binding site remains the focus of much interest regarding the discovery of new MTAs, [reviewed in [7,8]. We will first discuss the differences between species regarding their affinity for CBSIs and then between isotypes within a species. We then discuss how these two sources of variation are combined to support the repurposing of CBSIs in multiple contexts.

## 2. Inter-Species Differences and Identification of Species Selective CBSIs

Since the early twentieth century, colchicine has been known to induce mitotic arrest in mammals and other organisms because of the loss of mitotic spindle fibers (although the effect on the microtubule polymers (MT) that comprise these spindle fibers was not known at the time) [9]. It was also clear that the concentrations required for mitotic arrest varied tremendously among the species that responded to this agent.

Colchicine concentrations that induce mitotic arrest in mammalian cells are much lower (sub-μM) than those in plant cells (mM). Yeast and other fungal cells also require mM concentrations of colchicine or close analogs in order to induce mitotic arrest via the loss of MT [10]. These patterns are not shared with all other MTAs; for example, taxol shows similar activity in plant and animal cells [reviewed in [11]. These patterns of sensitivity to colchicine are reflected in published dissociation constants (Kd) for colchicine to tubulins from different sources: 0.1–1 μM (mammal), 5–30 μM (helminths), 5–1000 μM (plants), 200–2000 μM fungi [reviewed in [12]. Colchicine is still actively used in plant breeding to produce polyploid plant lines in order to enhance phenotypes, which require millimolar colchicine concentrations [13]. Notably, colchicine fairly specifically induces MT loss and consequent mitotic arrest in different cells over a 10^5^–10^6^-fold range of concentrations (from tens of nM for mammalian cells to tens of mM for plant and fungal cells).

It is now known that the range of species that avidly bind colchicine with a high affinity is restricted because the colchicine binding site is altered in many species compared to the canonical tubulin isotype that is found mainly in mammalian tissues (e.g., neuronal tubulin containing substantially class II and III beta tubulin). These changes in the structure of the binding cavity can lead to a reduced cavity volume [14] and/or changes in the polarity of the residues in the cavity wall [15].

A valuable application of the differences in CBS regarding their affinity for CBSI and ability to target different groups of organisms is the use of benzimidazole compounds such as benomyl, carbendazim, and others as systemic fungicides for house plants and in agriculture (as well as for human parasite infections). This action relies on the increased binding affinity of these compounds for fungal tubulin compared with that of plant (or animal) tubulin [16]. Fungal tubulin targeting was demonstrated via direct binding assays [17] and a study of resistance-conferring mutations in β-tubulin [18].

Colchicine binding site inhibitors have proven helpful in discriminating between tubulins from mammals and tubulins from medically relevant parasites, thereby providing a widely used therapy against helminths, nematodes, fungi, and protozoal parasites [19]. This species selectivity has been shown to be due to differential binding to tubulins from different sources [20]. 

## 3. Inter-Isotype Differences and Identification of Isotype-Selective MTAs

Different isotypes of tubulin from one organism can have more similar affinities for CBSIs than tubulins from organisms from different kingdoms of life. However, there are still differences in the binding affinity between isotypes that result from changes in the binding cavity that are similar to those found between different species. These differences between isotypes may prove to be useful therapeutically in order to treat conditions that preferentially rely on different isotypes of tubulin. Here we review a few examples of this that have been noted.

The hematopoietic-specific class VI β-tubulin isotype that is produced by the TUBB1 gene is the most divergent of the β-tubulin isotypes and shows several selectivities for CBSIs. One such selectivity is its resistance to 2-methoxyestradiol (2ME) binding, a low-potency inhibitor of tubulin polymerization, angiogenesis, and metastasis. This resistance results from the presence of an isoleucine in place of the valine in position 236 (V236) at the colchicine binding site, found in all other beta isotypes. This isoleucine results in some protection from 2ME-induced myelosuppression. This makes 2ME the first isotype-targeted chemotherapeutic, albeit targeted by exclusion [21]. The further development of this compound has led to the discovery of new derivatives with increased potency [22]. 

This same isotype (TUBB1) demonstrates binding that is not shared by other beta isotypes, in addition to the non-binding (exclusion) just discussed. Purified tubulin containing TUBB1 β-tubulin (from chicken erythrocytes) has shown binding and polymerization inhibition from some but not all anti-helminthic benzimidazoles, while mammalian brain tubulin did not under similar conditions. Tubulin purified from mammalian and chicken brains contains several β-tubulin isotypes, such as TUBB2 (class II) and TUBB3 (class III), but omits TUBB1 (class VI). The drugs albendazole, oxibendazole, and carbendazim have shown no binding to brain tubulin under assay conditions, but show a low μM K_d_ for chicken erythrocytes tubulin [23]. 

**Figure 1:**
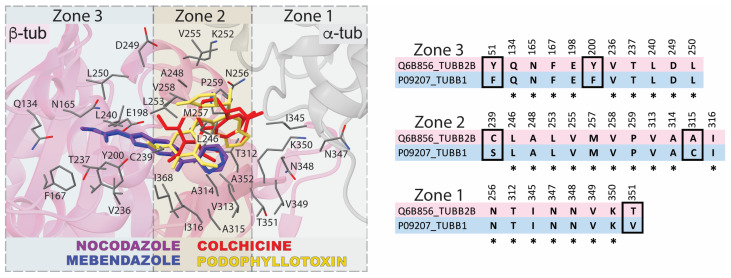
Tridimensional structure and sequence of the β-tubulin isotype TUBB2B colchicine binding site (CBS). Several drugs bind to TUBB2B but show differences in their localization and orientation inside the CBS. (**Left panel**): the drugs colchicine (red) and podophyllotoxin (yellow) bind to CBS zones 1 and 2, which are closer to the surface of TUBB2B than zone 3. The benzimidazole drugs nocodazole (blue) and mebendazole (purple) bind to CBS zones 2 and 3 (the two compounds nearly overlap in the figure). (**Right panel**): the sequence alignment of the amino acids corresponding to zones 1, 2, and 3 of TUBB2B, aligned with the corresponding sequence of hematopoietic β-tubulin isotype TUBB1. The residues enclosed in black are different between the two β-tubulin isotypes. The structural superposition of αβ-tubulin bound to the drugs colchicine (4O2B.PDB), podophyllotoxin (1SA1.PDB), nocodazole (7Z2P.PDB), and mebendazole (7OGN.PDB) was performed using ChimeraX-1.5 [25].

**Figure 2:**
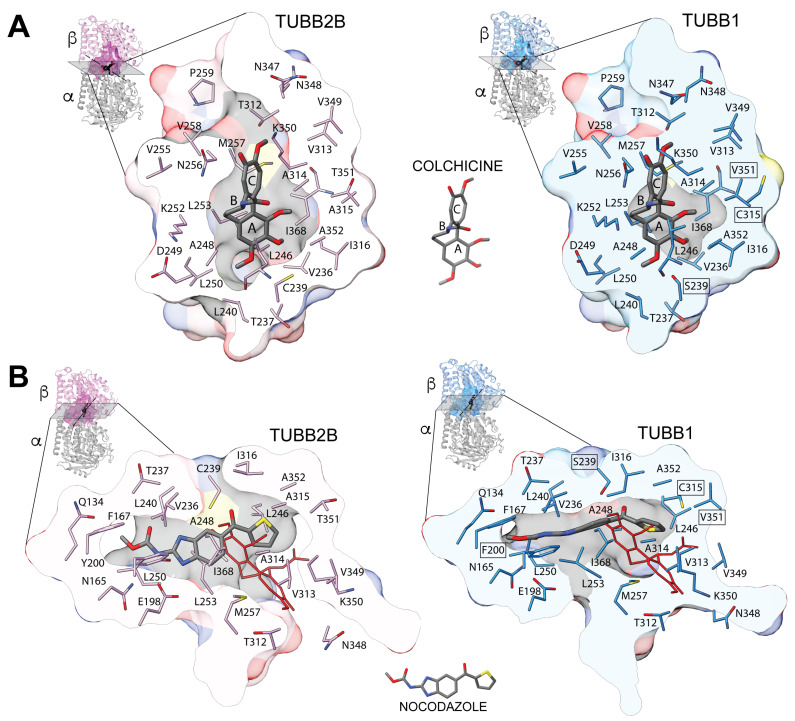
Structural superposition of the β-tubulin isotypes TUBB2B and the TUBB1 colchicine binding sites. (**A**) the size of the CBS cavity observed for the colchicine-bound TUBB2B (top left, gray) is more extensive than for TUBB1 (top right, gray). The estimated molecular volume of the colchicine molecule is 334 Å^3,^ and the CBS cavity volume of TUBB2B is 780 Å^3^. The structural model of TUBB1 shows a CBS cavity that is too small to dock a colchicine molecule (shown for reference), and it could not be found using the same protocol applied to TUBB2B2 with CB-Dock2 [26]. The TUBB1 structural model predicts the steric interference of K350 over colchicine ring C and of L246 over ring A. (**B**) the benzimidazole drug nocodazole binds to the CBS of TUBB2B, occupying a different zone than that of colchicine (red, included for reference). The corresponding cavity of TUBB1 can accommodate a nocodazole molecule using AutoDock vina [27]. Still, the molecule’s orientation is different than that of TUBB2B (colchicine is shown in red for reference), resulting in a lower predicted affinity score. The crystal structures of the colchicine-bound TUBB2B (4O2B.PDB, *Bos taurus*) and nocodazole-bound TUBB2B (7Z2P.PDB, *Bos taurus*) were used as a template in order to model the CBS of the tubulin isotype TUBB1 (*Gallus gallus*) using AlphaFold [28]. The alpha carbon backbone of the TUBB1 model was structurally aligned with TUBB2B, leaving colchicine at the center. A clipping plane through the CBS was generated in order to illustrate the cavity surrounding the bound colchicine molecule in the TUBB2B crystal structure. The same clipping plane was applied to the aligned structure of TUBB1 with the colchicine molecule drawn on top. The same strategy was used to model the binding site of nocodazole.

The selectivity of benzimidazole drugs for TUBB1 over brain β-tubulin (e.g., TUBB2B) can be understood considering the colchicine binding site structure (Figure 1). The CBS structure of β-tubulin TUBB2B (main isotype in brain tissue) is defined as having three zones, 1, 2, and 3. These zones are broadly defined based on the ligands they bind and their position in the αβ-tubulin structure. Zone 1 is located at the interface with α-tubulin. Zone 2 is the main zone, is located entirely in β-tubulin, is internal to zone 1, and accommodates most of the structure of the ligands. Zone 3 is found deeper in the core of β-tubulin. Zones 1 and 3 are considered to be accessory zones because they are involved in stabilizing only smaller portions of the ligands [24]. Residues in zones 1 and 2 are more solvent-exposed and are found closer to α-tubulin (Figure 1). Residues belonging to zone 3 are primarily hydrophobic and are found in the β-tubulin core. A superposition of the brain tubulin isotype TUBB2B structure, with several bound CBSIs, shows colchicine and podophyllotoxin bound to zones 1 and 2. The benzimidazole drugs nocodazole and mebendazole are bound to zones 2 and 3 (i.e., located more deeply in β-tubulin). The predicted structure of the TUBB1 colchicine binding site (Figure 2), superimposed on TUBB2B, shows a CBS cavity that is too small to dock a colchicine molecule (dark gray in Figure 2). The conservation of CBS residues between the two tubulins is ~84%, with some substitutions in TUBB1 found very close to the colchicine molecule (TUBB2B > TUBB1; Y200 > F; C239 > S; A315 > C; T351 > V) (See Figure 1 for the sequence alignment and Figure 2 for the structural comparison). Therefore, some structural rearrangements in the TUBB1 CBS might occur in order to accommodate the drugs. In the case of nocodazole, a planar molecule compared to colchicine, the TUBB1 CBS cavity docks the molecule but with a different orientation, resulting in a lower docking score (Figure 2B); this is indicative of a different CBS structure compared to TUBB2B.

Another benzimidazole CBSI that shows isotype selectivity is thiabendazole. Thiabendazole has been shown to have potent vascular-disrupting capacities owing to its ability to target one beta isotype, TUBB8, in newly formed blood vessels. Binding to TUBB8 appears to depend on three residues: F167, E198, and F200, which are all in this configuration only in TUBB8, accounting for the isotype selectivity [29]. 

Plinabulin is not a benzimidazole but a synthetic analog of the natural compound phenylahistin, and is currently being investigated as a potential treatment for cancer and neutropenia (currently in phase 3 clinical trials) [30]. The selectivity of plinabulin towards TUBB2 over TUBB3 in neural tissue is based on its differential binding and residence time in the CBS of the two isotypes, and could be exploited in order to target cancer cells that are known to overexpress TUBB3 in response to treatment with MTAs, such as Taxol [31].

## 4. Repurposing

Benzimidazole anti-parasite CBSIs have shown activity against multiple cancers, both in mice and in humans, and consequently are the subjects of numerous studies aiming to repurpose these compounds for the therapy of human cancers [32,33,34,35]. The results of these studies have demonstrated their activity against brain tumors, which was first observed in mouse models of glioma and medulloblastoma being treated with mebendazole for worm infestations [36]. Subsequent studies have indicated the activity of these compounds against a variety of cancers, including brain, head and neck, breast, colorectal, lung, ovary, prostate, pancreatic, and liver, due to their involvement in multiple mechanisms, including microtubule dynamics, signal transduction, glycolytic/oxidative metabolic balance, glucose uptake, anti-angiogenesis, and others [37,38,39,40]. A number of benzimidazoles have shown anticancer activity, notably mebendazole [34,35], but also albendazole [38], flubendazole [32], fenbendazole [41], parbendazole [39], and others [33,39]. Their activity has been studied both as monotherapy and in combination with standard therapy [33,34]. In spite of these encouraging reports, concerns have been expressed regarding the limitations of these compounds; for example, their low bioavailability has been noted [42], and limitations regarding the design and power of the studies being cited in support of this activity are evident [43]. In addition, it has been noted that the generic profile of most of these compounds will present difficulties with regard to their commercial development [44].

## 5. Summary

Differences in tubulin sequences can alter tubulin’s response to MTAs in several ways. Sequence differences may, but need not, change a residue by directly abutting the ligand in the binding site. Changes distal to the binding site may have allosteric, through-protein effects that alter binding, such as by moving a part of the wall of the binding cavity inward, thus reducing the volume available for ligand occupancy [45,46]. Since tubulin microtubules are a dynamic system, changes that alter the stability of the MT polymers versus unpolymerized dimers may alter the biological effect of the compound. For example, destabilizing compounds are less effective if the intrinsic stability of the MT is increased by sequence changes, which can happen remotely from the binding cavity [47].

Thus, changes in the tubulin sequences between species or between isotypes within a species can alter the biological effects of an MTA via several and similar mechanisms: changing the wall of the ligand cavity in direct contact with the ligand, altering a residue that is distal to the ligand cavity that alters the cavity via allosteric changes, or altering the intrinsic stability of the MT versus the unpolymerized dimer. Any or all of these may provide opportunities for novel therapeutic intervention. 

## Data Availability

The data that were analyzed in this study are publicly available and referenced in the text.
